# The Usefulness of Diffusion Tensor Tractography in Diagnosing Neuropathic Pain: A Narrative Review

**DOI:** 10.3389/fnins.2021.591018

**Published:** 2021-03-26

**Authors:** Seoyon Yang, SuYeon Kwon, Min Cheol Chang

**Affiliations:** ^1^Department of Rehabilitation Medicine, Ewha Women’s University Seoul Hospital, Ewha Women’s University School of Medicine, Seoul, South Korea; ^2^Department of Rehabilitation Medicine, College of Medicine, Yeungnam University, Daegu, South Korea

**Keywords:** diffusion tensor imaging, diffusion tensor tractography, central post-stroke pain, trigeminal neuralgia, sciatica, headache

## Abstract

Diffusion tensor tractography (DTT) is derived from diffusion tensor imaging. It has allowed visualization and estimation of neural tract injury, which may be associated with the pathogenesis of neuropathic pain (NP). The aim of the present study was to review DTT studies that demonstrated the relationship between neural injuries and NP and to describe the potential use of DTT in the evaluation of neural injuries that are involved in the pathophysiological process of NP. A PubMed search was conducted for articles published until July 3, 2020, which used DTT to investigate the association between neural injuries and NP. The key search phrase for identifying potentially relevant articles was (diffusion tensor tractography AND pain). The following inclusion criteria were applied for article selection: (1) studies involving patients with NP and (2) studies in which DTT was applied for the evaluation of NP. Review articles were excluded. Altogether, 108 potentially relevant articles were identified. After reading the titles and abstracts and assessment of eligibility based on the full-text articles, 46 publications were finally included in our review. The results of the included studies suggested that DTT may be beneficial in identifying the pathophysiological mechanism of NP of various origins including central pain caused by brain injuries, trigeminal neuralgia, sciatica, and some types of headache. Further studies are needed to validate the efficacy of DTT in investigating the pathophysiology of other types of NP.

## Introduction

Neuropathic pain is a localized sensation of unpleasant discomfort that results from a lesion or a disease of the peripheral or the central somatosensory system (Zhang et al., 2020). Common symptoms of NP include abnormal sensations (dysesthesia) and pain from non-painful stimuli (allodynia). Patients often complain of spontaneous ongoing or shooting pain, with evoked amplified pain responses after noxious or non-noxious stimuli (Baron et al., 2010). There are many different types of NP. Some of the common syndromes of NP are stroke-related central pain, TN, sciatica, and some types of headache. Medication is often applied initially for the treatment of NP, but NP tends to be refractory to conventional analgesics such as non-steroidal anti-inflammatory drugs, antidepressants, and opioids. Interdisciplinary approach, including neuromodulation treatments such as rTMS, transcranial direct current stimulation, and deep brain stimulation, is often needed for the treatment of NP. Identifying the pathophysiological mechanism of NP is important, as it can lead to a more effective and specific mechanism-based treatment approach.

Brain magnetic resonance imaging identifies a specific lesion as spotty appearance, but it cannot visualize neural tract injury. Some patients suffer from NP without showing any abnormal findings on conventional MRI or computed tomography, but their symptoms may be induced by microscopic injury of the neural tracts (Boudier-Revéret et al., 2020). DTT is a recently developed three-dimensional reconstruction technique of DTI. It shows fiber bundles with connectedness of voxels having similar diffusion properties (Oh et al., 2018). It is commonly applied in neurosurgical settings to avoid damage to surrounding vital neural pathways and to guide deep brain stimulation electrodes toward their targets (Zhang et al., 2020). DTT allows a quantitative analysis of healthy and pathological nerves, myelin sheaths, and muscles and enables the assessment of neural tract injuries at a microscopic level (Chianca et al., 2017).

Neuropathic pain can develop following nerve injury after neurons are injured. It occurs when harmful changes have occurred along the nociceptive and the descending modulatory pathways in the CNS (Cohen and Mao, 2014). Clarification of the pathophysiological mechanism of NP may provide clues for effective treatment modalities to treat patients suffering from NP. Several studies have been conducted to investigate the injuries of neural tracts in patients with NP. The aim of the present study was to review DTT studies that demonstrated the relationship between neural injuries and NP, and to describe the potential use of DTT in the evaluation of neural tract injuries that are involved in the pathophysiological process of NP.

## Methods

**FIGURE 1:**
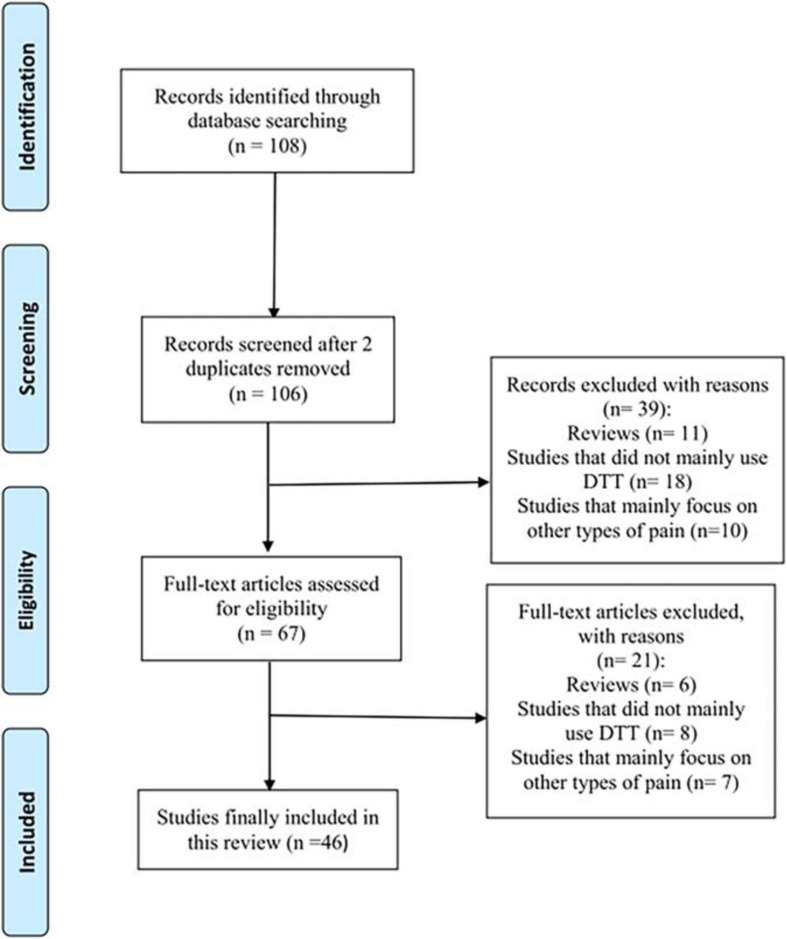
Flow diagram of the study selection process. DTT, diffusion tensor tractography.

**FIGURE 2:**
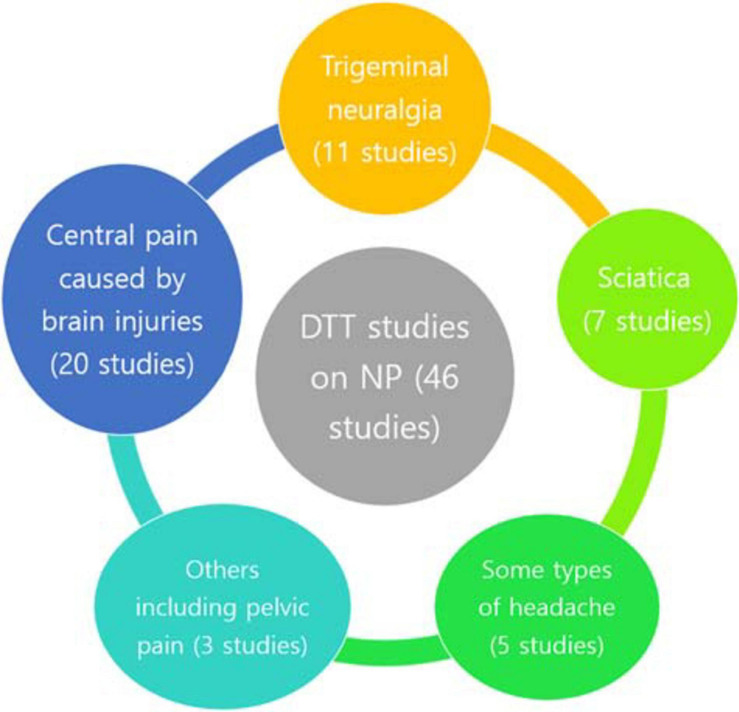
Diffusion tensor tractography studies on NP. DTT, diffusion tensor tractography; NP, neuropathic pain.

Studies that investigated neural injuries in various types of NP using DTT were searched. We searched the MEDLINE database (PubMed) for relevant studies published until July 3, 2020. The key search phrase for identifying potentially relevant articles was (diffusion tensor tractography AND pain). The following inclusion criteria were applied for the selection of articles: (1) studies involving patients with NP and (2) studies in which DTT was applied for the evaluation of NP. This review was limited to studies involving humans with NP. The relevant studies were selected according to the flow diagram shown in Figure 1. Altogether, 108 potentially relevant articles were identified. After reading the titles and abstracts and assessing the eligibility based on the full-text articles, 46 publications were included in our review. Studies eligible for the review included 20 studies on central pain caused by brain injuries, 11 studies on TN, 7 studies on sciatica, 5 studies on headache, 1 study on thoracic outlet syndrome, and 2 studies on CPP (Table 1 and Figure 2).

**TABLE 1:** Characteristics of the included studies.

S. No.	First author	Year	Number of patients	Duration to DTT	Location of lesion	Characteristics of pain	Results
**Central pain**							
1	Seghier	2005	1	4–5 years	ICH (thalamus and internal capsule)	NA	DTT revealed a reduction in the fiber density of the lateral TCT, which was presumed to be the STT.
2	Goto	2008	17	5.1 years (1–8.8 years)	Supratentorial stroke	Refractory pain lasting more than 6 months	The rTMS-effective group showed higher delineation ratio of the CST and the TCT than the rTMS-ineffective group.
3	Hong	2010	30	20 months (5–48 months)	ICH (corona radiata and basal ganglia)	NA	TV was decreased without any changes in the FA or MD values in the CPSP group, indicating partial STT injury.
4	Hong	2012	52	18.8 months (5–46 months)	ICH (corona radiata, basal ganglia, and thalamus)	NA	Number of patients with partial STT injury was higher than the number of patients with complete STT injury among patients with CPSP.
5	Jang	2013	1	1 month	TBI (thalamus and ventral posterolateral nucleus)	Persistent tingling sensation and pain	The FA values of the left STT at the thalamus were decreased, indicating injury of the left ventral posterolateral nucleus.
6	Hirayama	2014	1	2 weeks	Stroke (right parietal operculum and insula)	Sensation of pain decreased markedly in multiple regions	The operculo-insular lesion disrupted the ipsilateral thalamo-S2 projections when compared with the intact side.
7	Jang	2014	1	2 years	TBI (axonal injuries in both the cingula and in the optic radiation)	Pain in multiple regions	Both the STTs of the CPSP patient were thinner in gross appearance with decreased FA and TV
8	Kim	2015	22	3–28 months	Mild TBI	NA	Decreased FA and TV and increased MD were observed in the pain group when compared with the control group.
9	Jang	2016	1	1 month and 9 months	Mild TBI	Pain in the hand and foot with constant tingling	On 1-month DTT, partial tearing was observed in both the STTs. Both partially torn STTs had atrophied on 9-month DTT.
10	Jang	2017	2	4–5 days	Mild TBI	Pain in multiple regions with tingling	Partial tearing of the STTs was observed in both patients.
11	Jang	2017	1	1 month and 3 years	Corpus callosum hemorrhage	Pain in the upper and lower extremities with tingling	Disruption of transcallosal fibers in the genu and the isthmus of the corpus callosum was observed after 1 month and thinning of the STT was observed 3 years later.
12	Jang	2017	1	4 weeks	Mild TBI	Pain in the leg, throbbing and cold pain	The CST showed partial tearing at the subcortical white matter level. Narrowing and partial tearing were observed in both the STTs.
13	Jang	2017	5	11 days (10–13 days)	Cerebral infarction (corona radiata and thalamus)	Pain including electric shock-like sensation	FA and TV values were decreased with STT injury in the affected hemisphere in all patients.
14	Choi	2018	12	2.6 months	Mild TBI	Pain including electric shock-like sensation	FA and TV values of the STTs were decreased with partial tearing of the STTs in all patients.
15	Jang	2018	1	2 weeks and 14 months	Thalamic hemorrhage	Pain in the arm and leg with tingling and cold sensation	Partial tearing and thinning was observed in the left STT.
16	Lee	2018	1	2 months after onset	Stroke (left MCA infarct)	Painful range of motion	Decreased number of CST and STT fibers were observed in both unaffected and affected hemispheres.
17	Jang	2019	1	9 years	TBI (whiplash injury)	Burning pain in the arms and legs with constant tingling	Both the STTs showed marked narrowing and partial tearing. Partial tears were observed in both the CSTs.
18	Jang	2019	5	2 days to 14 months	Mild TBI	Pain including electric shock-like sensation	The FA values were decreased and the STT showed partial tearing in at least one hemisphere.
19	Jang	2019	1	4 years	Mild TBI	Intermittent, squeezing, and warm creeping-like pain in the abdomen	The upper portion of the STTs in both hemispheres showed partial tearing.
20	Jang	2019	1	2 months	Mild TBI	Pain with burning sensation	Partial tearing of the CST was observed at the subcortical white matter in both hemispheres. Tearing was much more severe in the left CST.
**Trigeminal neuralgia**							
21	Hodaie	2012	5	NA	Classic TN with most common distribution along V2	Medically intractable TN pain	Radiosurgery resulted in a 47% drop in the FA values at the target, demonstrating highly focal changes after treatment.
22	Wilcox	2015	21	5.5 years	Painful TN according to the Liverpool criteria	Unilateral or bilateral pain	No significant differences were observed in diffusivity of pathways between TN patients and controls.
23	Chen	2016	20 (10 TN and 10 MS-TN)	NA	Unilateral TN versus MS-TN	NA	DTT showed a difference in microstructural changes along the CN V between the TN group and the MS-TN group.
24	Burkett	2017	17	NA	NA	NA	The descending tract of the trigeminal nerve was visualized in all patients.
25	Hayes	2017	37	0.5–10 years	NA	Extreme unilateral pain	The cingulum and the medial forebrain bundle were altered in patients with TN. The posterior cingulum and the MFB-VTA also showed unilateral differences.
26	Moon	2018	14	0.5–10 years	Mostly V2 and V3 distribution	Unilateral TN	Patients with TN showed decreased FA and increased MD and RD on the affected side.
27	Tohyama	2018	37	1.5–25 years	V1, V2, V3 were affected.	Diagnosis of classic TN	Long-term responders to GKRS showed lower FA and higher RD and MD in the affected nerve.
28	Li	2019	18	Median 3 years	Mostly V2 and V3 distribution	Medically intractable MS-TN pain	Preoperative assessment of the trigeminal pathway was a better indicator of GKRS response.
29	Rutland	2019	10	1–144 months	V2 was affected in all patients.	NA	Topographical analysis revealed decreased FA and elevated diffusivity along the entire anatomical S1 arc in patients with TN.
30	Choi	2020	1	7 years	Pontine hemorrhage with injury of the CN V	Facial pain with tingling and cold sensation	The affected CN V was discontinued at the anterior margin of the pons when compared with the unaffected side.
31	Yoshida	2020	1	20 years	Venous malformation in the trigeminal nucleus	Typical and severe TN	DTT revealed that the venous malformation was located in the trigeminal nucleus of the middle cerebellar peduncle.
**Sciatica**							
32	Chuanting	2014	20	NA	L4-5 and L5-S1 LDH	Unilateral radicular pain	The FA value in compressed spinal nerve roots was lower than that on the unaffected side.
33	Oikawa	2015	34	NA	LDH or lumbar SS with/without FS	NA	More abnormalities were observed in patients with lumbar SS and especially in patients with FS. The mean FA of the entrapped nerves was low.
34	Shi	2015	75	4–12 months	LDH or FS	Unilateral radicular leg pain	Abnormalities were observed in 46 cases (88.5%) with symptomatic L4 nerve roots and in 21 cases (91.3%) with symptomatic S1 nerve roots.
35	Eguchi	2016	1	5 years	Bilateral L5 FS	LBP with pain in both legs	Interruption of fibers was observed at the L5 vertebral foramen on tractography.
36	Wu	2016	34	1.3–4.4 months after the surgery	Unilateral S1 LDH compressing the nerve root	Radicular pain	The FA value of the compressed nerve roots before surgery was lower than that after surgery, but no difference was observed after the surgery.
37	Wu	2016	40	0.3–6 months	Unilateral L5-S1 LDH compressing the nerve root	Radicular pain	The mean FA value of the compressed lumbar nerve roots was significantly lower than that on the unaffected side.
38	Shi	2020	36	NA	Unilateral nerve root compression with L4-5 or L5-S1 LDH	LBP with leg pain and tingling	Abnormalities in the symptomatic nerve tracts were observed in the middle or the distal sub-regions in 33 cases (91.7%).
**Headache**							
39	Chou	2014	17	6–13 months	NA	Unilateral cluster headache attacks	Tractography showed highly consistent anatomical connections between altered areas of the brain and the hypothalamus.
40	Chong	2015	23	Mean 18 years	Migraine	Episodic or chronic migraine	Patients with migraine showed increased MD and RD in the anterior thalamic radiations and in the CST, with no differences in FA.
41	Coskun	2017	2	5 months and 6 years	SUNCT	Severe, excruciating pain in the periorbital area	Neurovascular compression of the CN V by the superior cerebellar artery was observed with decrease in the FA value on the affected side.
42	Jang	2019	2	5 months and 10 months	Headache after mild TBI	Constant tingling and intermittent stabbing pain	The STTs showed narrowing in both the hemispheres and discontinuations at the subcortical white matter were observed in both the hemispheres in patient 2.
43	Wang	2020	26	NA	NA	NA	The FA values of the GON and the cervical DRG on the symptomatic side were lower than those on the asymptomatic side.
**Other**							
44	Manganaro	2014	30	NA	Endometriosis and/or endometriotic nodules	Moderate to severe chronic pelvic pain	Bilateral abnormalities of S1, S2, and S3 were observed including disorganized appearance and decrease in the FA values in the nerve roots.
45	Magill	2015	1	10–15 years	Neurogenic thoracic outlet syndrome	Weakness with tingling	Preoperative tractography revealed the compression caused by the scalene muscle on C6 and C8 nerve roots.
46	Porpora	2018	56	36 months	Endometriosis and/or adenomyosis	Non-cyclic pelvic pain	Abnormalities in microstructure reconstruction with fiber disorganization and loss of unidirectional course were observed in 44 patients (66.7%).

*CST, corticospinal tract; DRG, dorsal root ganglion; FA, fractional anisotropy; FS, foraminal stenosis; GKRS, Gamma knife radiosurgery; GON, greater occipital nerve; ICH, intracerebral hemorrhage; LDH, lumbar disk herniation; LBP, lower back pain; MCA, middle cerebral artery; MD, mean diffusivity; MFB-VTA, medial forebrain bundle near the ventral tegmental area; MS, multiple sclerosis; NA, not available; RD, radial diffusivity; rTMS, repetitive transcranial magnetic stimulation; SS, spinal stenosis; STT, spinothalamic tract; SUNCT, short-lasting unilateral neuralgiform headache attack with conjunctival injection and tearing; TBI, traumatic brain injury; TCT, thalamocortical tract; TN, trigeminal neuralgia; TV, tract volume; V1, ophthalmic division of the trigeminal nerve; V2, maxillary division of the trigeminal nerve; V3, mandibular division of the trigeminal nerve.*

A combined ROI method, which reconstructs the neural fibers passing more than two ROI areas, is usually used in DTT studies for reconstruction of the neural tracts (Jang et al., 2019b). The entire neural tract can be evaluated in terms of DTT parameters. Commonly used DTT parameters are FA, MD, and TV. FA reflects the degree of directionality of water diffusion. It is used to estimate the degree of directionality and the integrity of white matter microstructures including axons, myelin, and microtubules. MD reflects the degree of water diffusion. In conditions such as vasogenic edema or axonal damage, it exhibits a tendency to increase. TV indicates the number of voxels included in the neural tract. Neural injury is assumed to be present when there are decreases in the values of FA and TV and increases in the value of MD (Assaf and Pasternak, 2008; Jang et al., 2019b). Some limitations of DTT should also be considered. DTT may underestimate the fiber tracts. It may show the gross fiber architecture, but cannot demonstrate the functional or synaptic connections. The results of DTT may differ according to the operator, as the fiber tracking technique is operator dependent. Complete reflection of the fiber architecture may be hampered in regions where the fibers are complex and where they cross each other.

## Diffusion Tensor Tractography for Diagnosis of Various Types of Neuropathic Pain

### Central Pain Caused by Brain Injuries

Central pain is one of the most common types of NP. Patients with central pain suffer from various symptoms such as tingling, numbness, burning, chilling, hyperpathia, itching, and abnormal sensations. It is caused by a lesion or dysfunction of the somatosensory pathways in the CNS, which most commonly involves the brain and spinal injuries (Yang and Chang, 2020). Importantly, central poststroke pain (P) is one of the most common sequelae of stroke, which is caused by cerebrovascular injury to the somatosensory pathway in the brain (Akyuz and Kuru, 2016). The suggested pathophysiological mechanisms of CPSP include central sensitization, changes in neural excitability due to disinhibition, alteration of the somatosensory function, changes in the thalamic function, and inflammation of the involved neural tract. Neural structures associated with CPSP include the STT, medial lemniscus pathway, thalamus, and cerebral cortex (Klit et al., 2009; Jang et al., 2019b).

Twenty studies were identified, which used DTT to investigate the presence of neural tract damage in patients with central pain that occurred after brain injuries. In a case report by Seghier et al. (2005), DTT was used to visualize the reduction in fiber density of the lateral thalamocortical tract. Goto et al. (2008) reconstructed the CST and the thalamocortical tract in chronic stroke patients suffering from CPSP. Some patients with CPSP showed interruption of the STT pathway. Moreover, lesions of the thalamocortical tract correlated with the efficacy of rTMS (90% intensity of the resting motor threshold, 10 trains of 10-s, 5-Hz TMS pulses with 50-s intervals). Hong et al. (2010) used DTT to demonstrate STT injuries in patients with CPSP after ICH. Patients were divided into the CPSP group (16 patients) and the non-CPSP group (14 patients). Two ROIs were used to reconstruct the STT. MLP and fiber tracking were performed using a probabilistic tractography method. The results showed that TV was decreased without changes in the FA or MD values in the CPSP group. This study suggested that the presence of partial STT injury was associated with the development of CPSP. Hong et al. (2010) reported that the prevalence of CPSP in patients with ICH was higher in cases of partial STT injury when compared with that in cases of complete STT injury. Recently, Jang et al. (2017a, 2018) reported that patients with ICH who suffered from CPSP exhibited partial tearing and thinning of the STT. Furthermore, several case reports (Hirayama et al., 2014; Jang et al., 2017b) showed that disconnection of the brain pathway could lead to CPSP after acute infarction. Jang et al. (2017b) reported five patients with CPSP after cerebral infarction who showed decreased FA and TV when compared with the control group. Affected STTs passed through an adjacent part of the infarct such as the corona radiata and the thalamus, suggesting that STT injury may trigger the development of CPSP. Interestingly, a previous report described a patient who had CRPS following an ischemic stroke at the left middle cerebral artery territory (Lee et al., 2018). DTT showed a decreased number of CST and STT fibers.

Regarding central pain following TBI, several studies have demonstrated the association between neural tract damage and the development of central pain. Seo and Jang (2013, 2014) reported cases with abnormal STT findings detected using DTT, which showed that the STTs were thinner, and the FA and TV values were decreased, in patients with central pain. A retrospective study was conducted by Kim et al. (2015), which enrolled 32 patients with mild TBI. Patients were divided into two groups based on the presence or absence of central pain (22 patients in the pain group and 10 patients in the non-pain group). The STTs were reconstructed by selection of fibers passing through the ROIs. A seed ROI was placed in the STT of the posterolateral medulla. The first target ROI was around the ventral posterolateral nucleus of the thalamus, and the second target ROI was placed at the primary somatosensory cortex (S1). Decreased FA and TV and increased MD of the STTs were observed in patients who had central pain when compared with the control group, suggesting that STT injury was related to the occurrence of central pain. Cases involving patients with mild TBI and central pain were continuously reported in 2016 and 2017 (Jang and Kwon, 2016; Jang and Lee, 2016, 2017). These studies showed that partial tearing of the STTs was associated with the presence of central pain. Choi et al. (2018) used DTT to demonstrate partial STT injury in patients with mild TBI who had chronic central pain. A seed ROI was placed on the posterolateral medulla, and a target ROI was placed at the S1. Six patients who were treated with high-frequency rTMS (10 Hz with 90% intensity of the motor threshold and 1,000 pulses) reported reduction in central pain, suggesting that rTMS can be used to manage chronic central pain. Jang et al. (2019a) used DTT to demonstrate several cases with neural tract damage in patients with central pain. DTT was used to demonstrate STT injury in relation to central pain (Jang et al., 2019a; Jang and Lee, 2019) and to find out STT injury in patients who had atypical type of central pain including abdominal pain (Jang et al., 2020). Interestingly, CST damage was revealed using DTT in patients with CRPS following mild TBI (Jang and Seo, 2020). These studies showed that DTT was useful in detecting STT injuries in patients with central pain after TBI.

### Trigeminal Neuralgia

Trigeminal neuralgia is the most common type of chronic neuropathic facial pain disorder. The trigeminal nerve or the CN V comprises of the ophthalmic, maxillary, and mandibular branches. It controls the sensory and motor functions of the face. TN is characterized by recurrent attacks of sudden-onset, intermittent, highly intense, and shock-like pain over the area of distribution of the trigeminal nerve branches (Elias and Burchiel, 2002). TN is diagnosed clinically by transient or paroxysmal episodes of pain caused by neurovascular compression in the absence of other diseases. In classic TN, patients are often unable to identify the inciting event. In symptomatic TN, patients exhibit identifiable vascular compression of the trigeminal nerve caused by a tumor, multiple sclerosis, or an arteriovenous malformation (Jones et al., 2019). Brain imaging studies including MRI, DTI, and DTT have been performed to investigate the pathophysiology of TN.

In 2012, a DTT study on TN was conducted to investigate whether changes can be observed in the target area after radiosurgery for the treatment of TN (Hodaie et al., 2012). This study enrolled five patients with TN, and tractography was used to delineate the course of cranial nerves and to detect changes in the CN V after focal radiosurgery for the treatment of TN. In 2015, DTT was used to assess the integrity of ascending pain pathways in 21 patients with painful TN (Wilcox et al., 2015). DTI analysis revealed that orofacial NP was associated with significant increase in FA, decrease in MD, and decrease in the regional gray matter volume within the spinal trigeminal nucleus. Chen et al. (2016) used tractography to examine the differences in CN V microstructure between classic TN and TN secondary to multiple sclerosis (MS-TN). Patients with classic TN (10 patients) showed increased FA in the ipsilateral cisternal segment and decreased FA in the ipsilateral root entry/exit zone, whereas patients with MS-TN (10 patients) showed decreased FA in the ipsilateral perilesional segments. This study demonstrated that DTT technique can be used to distinguish different diffusivity changes in CN V segments in cases of classic TN and MS-TN. Hayes et al. (2017) used tractography to investigate the impact of TN on white matter tracts in 37 patients with TN. The results showed that the cingulum and the medial forebrain bundle were altered in patients with TN. Moreover, the posterior cingulum and the medial forebrain bundle near the ventral tegmental area showed unilateral differences between right- and left-sided patients. Moon et al. (2018) used tractography to show that the affected sides of 14 patients with TN showed significantly decreased FA, increased MD, and increased RD when compared with the unaffected sides.

Additionally, two studies showed that tractography can be used to illustrate microstructural changes in the affected CN V before and after GKRS, which is considered an important treatment modality for TN (Tohyama et al., 2018; Li et al., 2019). Changes in FA after GKRS were visualized at the radiosurgical target in long-term responders with classic TN (Tohyama et al., 2018). Decreased myelination at the proximal pontine segment was correlated with poor response to treatment in patients with MS-TN (Li et al., 2019). In 2019, microstructural alterations were observed in the thalamic-somatosensory tracts of patients with TN (Rutland et al., 2019). Moreover, a decrease in FA of the thalamic-somatosensory tract ipsilateral to the site of neurovascular compression was observed, while MD and RD were increased ipsilaterally in 10 patients with TN when compared with the control group. Recently, Choi et al. (2020) reported a case showing discontinuation of the CN V at the anterior margin of the pons on the side ipsilateral to the one with neuralgic pain after damage from pontine hemorrhage. In another case, DTT elucidated the presence of venous malformation located in the trigeminal nucleus of the middle cerebellar peduncle in intractable TN (Yoshida et al., 2020).

### Sciatica

Sciatica is pain that runs from the lower back down the leg. However, the term has been used widely for a variety of back and leg symptoms (Ropper and Zafonte, 2015). Injury of the lumbosacral nerve roots is most frequently caused by degenerative lumbar disease such as disk herniation, spinal stenosis, and degenerative lumbar spondylosis (Shi et al., 2020). Radicular leg pain is often described as sharp, aching, throbbing, or burning. The causes of radiating leg pain are not fully understood, but the mechanism of sciatica is presumed to be associated with inflammation or damage to the nerve root or the sensory ganglion (Ropper and Zafonte, 2015). Several studies have demonstrated that DTI with tractography is useful in the evaluation and visualization of peripheral nerves including the lumbar nerves.

Chuanting et al. (2014) enrolled 20 patients with L4–L5 and L5–S1 disk herniation who had unilateral radicular leg pain. Tractography was performed using the ROI method. Seed ROIs were placed at the level of the middle spinal body and the spinal disk. The results showed that the mean FA value of the compressed spinal nerve roots was significantly lower than that of the contralateral nerve roots. Oikawa et al. (2015) showed that among 34 patients who had lumbar degenerative disease, patients with lumbar spinal stenosis, especially the ones with foraminal stenosis, showed abnormalities on DTT. In the same year, Shi et al. (2015) suggested that DTT vividly demonstrated abnormalities in the symptomatic nerve tract. Abnormalities were observed in 67 (89.3%) out of 75 patients with radicular leg pain and numbness (Shi et al., 2015). In 2016, a study used DTI with tractography in patients with radicular pain and showed that the FA value of the compressed sacral nerve root was significantly lower than that on the unaffected side. Moreover, the FA value before surgery was significantly lower than that after surgery for compressed sacral nerve root (Wu et al., 2016a, b). Another case report described a case of a patient with bilateral L5 lumbar foraminal stenosis who showed an interruption of fibers on tractography at the L5 vertebral foramen (Eguchi et al., 2016). In 2020, a study by Shi et al. (2020) reported that DTT showed distinct abnormalities in the symptomatic nerve tracts in 33 patients (91.7%) who had compressed nerve roots.

### Headache Including Cluster Headache and Migraine

Primary headaches cause recurrent or persistent head pain, which can be debilitating, particularly in the chronic form (Mier and Dhadwal, 2018). The diagnosis of primary headaches relies on the history of no apparent underlying cause. Various types of primary headaches include migraine, CH, and SUNCT. The etiology of secondary headaches is unclear. The understanding of the pathophysiology of various types of headaches has continuously evolved and focuses on neuroimaging techniques to study altered sensory processing and brain pathways including neural tract injuries (Mier and Dhadwal, 2018).

Cluster headache presents with periodic and severe unilateral periorbital pain, which can be described as stabbing, squeezing, or burning. Chou et al. (2014) performed tractography on 17 CH patients, which revealed highly consistent anatomical connections between the altered areas (cerebellar tonsil or medial frontal gyrus) and the hypothalamus. This study suggested that descending projections of the hypothalamus may be involved in initiating autonomic responses and CH. Migraine usually presents as unilateral attacks of throbbing head pain with an episodic presentation, which may continue in a chronic or a refractory form. Probabilistic tractography was performed by Chong and Schwedt (2015) in 23 adults with migraine. Migraine patients showed increased MD and RD in the left anterior thalamic radiations, the left CST, and the right inferior longitudinal fasciculus tract, with no differences in FA. Interestingly, a positive correlation was observed between years lived with migraine and MD in the right anterior thalamic radiations and the left CST. SUNCT is one of the trigeminal autonomic cephalagias, which presents as attacks of unilateral head pain and cranial autonomic symptoms. A case report involving two patients with SUNCT showed that tractography was useful in identifying structural changes in the CN V secondary to neurovascular compression (Coskun et al., 2017).

Diffusion tensor tractography can be also used to identify the pathophysiology of headache with central pain caused by brain injury. In 2019, a case report showed that STT injury may be associated with posttraumatic headaches following TBI (Jang and Seo, 2019). In 2020, a study on cervicogenic headache, which is classified as a secondary headache arising from degenerative cervical spine disorders, was conducted using tractography in 26 patients (Wang et al., 2020). Tractography of the greater occipital nerves revealed a decrease in FA on the symptomatic side when compared with the asymptomatic side. DTT was useful in elucidating the complex pathophysiology in various types of headaches.

### Others

In addition to the aforementioned disorders and conditions, DTT has been used to assess microstructural changes in other painful conditions such as thoracic outlet syndrome and pelvic pain. Interestingly, Magill et al. (2015) used tractography to visualize compression caused by the scalene muscle on the C6 and C8 nerve root in a patient with neurogenic thoracic outlet syndrome who suffered from right arm weakness and tingling sensation. Endometriosis is a gynecologic disorder associated with pain symptoms including non-cyclic CPP, dysmenorrhea, and dyspareunia. Manganaro et al. (2014) enrolled 30 patients with endometriosis and moderate to severe CPP. DTI with tractography revealed that most of the patients displayed bilateral abnormalities of S1, S2, and S3 including an irregular and disorganized appearance with significant decrease in the FA values in the S1, S2, and S3 roots. Porpora et al. (2018) enrolled 76 patients with endometriosis and evaluated the abnormalities of the sacral root using DTI tractography. Their study also revealed irregularities in the sacral root microstructure associated with non-cyclic CPP.

## Conclusion

The diagnosis of NP often relies on the presence of a clinical symptom, and there is no definite diagnostic tool that accurately identifies the exact underlying pathophysiology of NP. Studies in our review showed the possible role of DTT in demonstrating neural injuries in patients with various types of NP. Based on the results of studies included in our review, we suggest that DTT may be an additive diagnostic method for visualizing neural injuries in patients with various types of NP. It is yet insufficient to conclude that DTT is a definite diagnostic tool for NP, but overall, DTT seems to have potential to be useful for defection of underlying pathophysiology of NP. NP may occur when neural tracts are injured; reductions in the fiber density and injuries such as tearing and thinning in neural tracts were observed in NP patients who were suffering from central pain caused by brain injuries, TN, sciatica, and various types of headaches. DTT appears to be useful for isolating neural tract injuries associated with pathologic conditions; it allows visualization of the neural tracts and demonstrates gross fiber architecture. It provides useful quantitative information about muscular tissue and peripheral nerves, and it can also be used to assess microstructural changes at the radiosurgical target to achieve long-lasting pain relief. In the future, DTT may become useful in selecting patients who may benefit from different treatments that target NP and in planning a more personalized therapeutic approach. In addition, DTT assessment can provide prognostic information and guide the clinical decision-making process in patients with NP. Future studies applying this new approach and involving larger patient populations are needed.

## Author Contributions

All authors listed have made a substantial, direct, and intellectual contribution to the work, and approved it for publication.

## Conflict of Interest

The authors declare that the research was conducted in the absence of any commercial or financial relationships that could be construed as a potential conflict of interest.

## Abbreviations

CH: cluster headache
CN V: fifth cranial nerve
CNS: central nervous system
CPP: chronic pelvic pain
CPSP: central post-stroke pain
CRPS: complex regional pain syndrome
CST: corticospinal tract
DTI: diffusion tensor imaging
DTT: diffusion tensor tractography
FA: fractional anisotropy
GKRS: Gamma knife radiosurgery
ICH: intracerebral hemorrhage
MD: mean diffusivity
MRI: brain magnetic resonance imaging
MS-TN: trigeminal neuralgia secondary to multiple sclerosis
NP: neuropathic pain
RD: radial diffusivity
ROI: region of interest
rTMS: repetitive transcranial magnetic stimulation
STT: spinothalamic tract
SUNCT: short-lasting unilateral neuralgiform headaches with conjunctival injection and tearing
TBI: traumatic brain injury
TN: trigeminal neuralgia
TV: tract volume.
